# Targeting Acute Myeloid Leukemia with 1,2,4-triazolo[4,3-b]pyridazine derivatives: a molecular docking, dynamics, and ADMET approach

**DOI:** 10.1007/s40203-025-00418-1

**Published:** 2025-09-17

**Authors:** Vincent A. Obakachi, Krishna K. Govender, Penny P. Govender

**Affiliations:** Department of Chemical Sciences, University of Johannesburg, Doornfontein Campus, P.O. Box 17011, Johannesburg, 2028 South Africa

**Keywords:** Acute Myeloid Leukemia, Mcl-1 inhibitors, 1,2,4-triazolo[4,3-b]pyridazine, Molecular docking, Molecular dynamics, ADMET profiling

## Abstract

Acute Myeloid Leukemia (AML) remains a critical therapeutic challenge, warranting the development of novel inhibitors targeting essential survival proteins such as Myeloid Cell Leukemia-1 (Mcl-1). In this purely computational study, a series of 1,2,4-triazolo[4,3-b]pyridazine derivatives were assessed for their potential as Mcl-1 inhibitors through molecular docking, molecular dynamics (MD) simulations, quantum chemical calculations, and ADMET profiling. Docking identified compounds **8f**, **8j**, **8k**, and **8l** as promising candidates, with **8l** exhibiting the most favorable binding free energy (ΔG_bind_ = − 58.96 kcal/mol). Docking-derived inhibition constants (*Ki*) revealed that **8f**, **8j**, **8k**, and **8l** had *Ki* values of 0.31 µM, 0.32 µM, 0.35 µM, and 0.34 µM, respectively, while Sunitinib showed a slightly weaker *Ki* of 0.36 µM. MD simulations demonstrated increased structural stability of the protein–ligand complexes, with RMSD values ranging from 1.68 Å (8f) to 1.90 Å (**8j**), compared to the unbound APO structure (2.12 Å). Compound **8l** maintained a low RMSD (1.71 Å) and favorable flexibility profile (RMSF = 0.89 Å), comparable to Sunitinib (RMSF = 0.76 Å). DFT analysis highlighted 8l’s high electronic reactivity, with a HOMO–LUMO gap of 3.18 eV in DMF. Although prior experimental studies confirmed **8l** potent anti-AML activity (IC₅₀ = 1.5 µM), ADMET predictions revealed pharmacokinetic limitations, including low solubility and permeability. These findings position compound **8l** as a compelling lead candidate for AML therapy and provide a strong foundation for future optimization aimed at improving its pharmacokinetic profile and dynamic stability. To substantiate these computational findings, we will initiate experimental validation studies involving Mcl-1 binding assays and cytotoxicity evaluation in AML cell lines.

**Graphical abstract:**

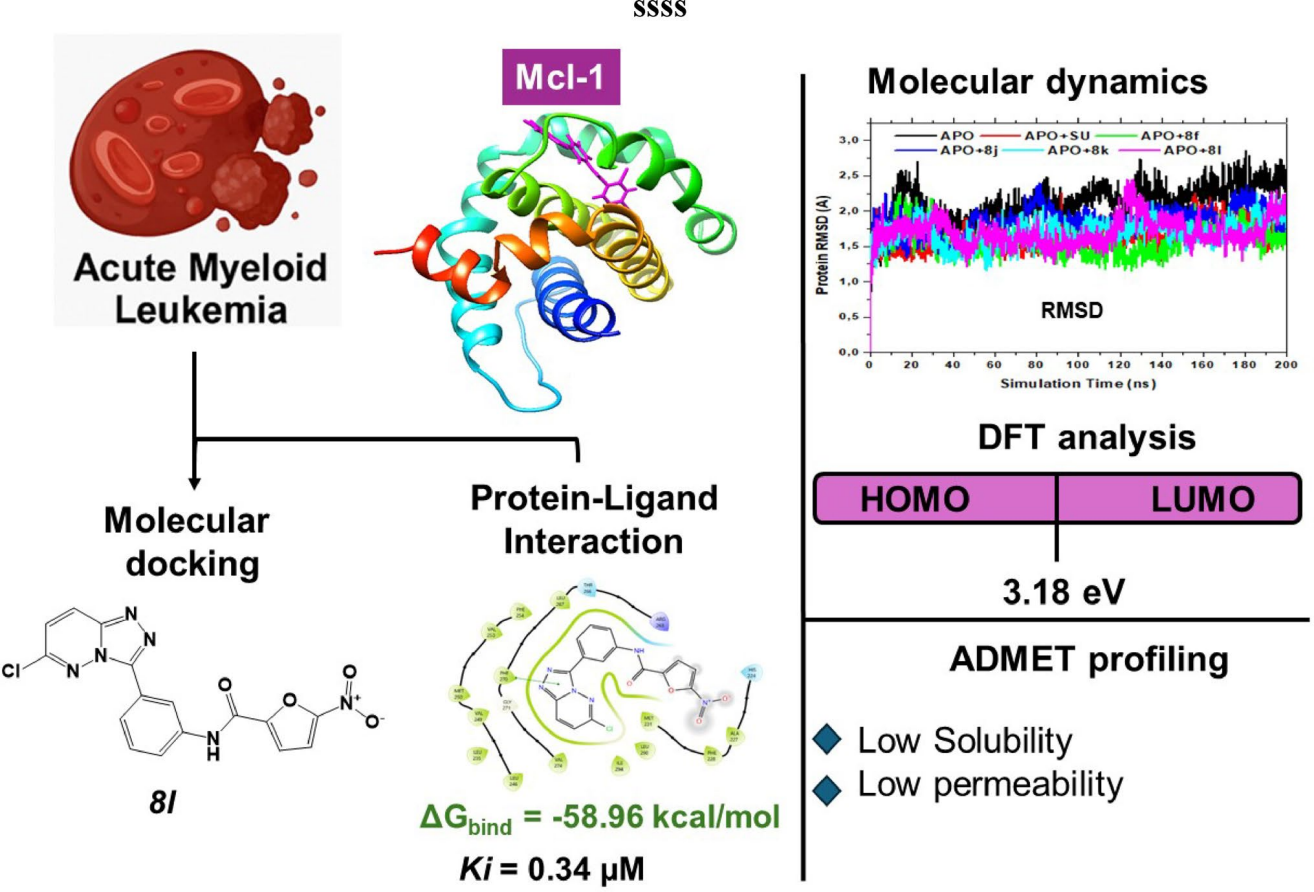

**Supplementary Information:**

The online version contains supplementary material available at 10.1007/s40203-025-00418-1.

## Introduction

Cancer remains a leading cause of morbidity and mortality worldwide, with Acute Myeloid Leukemia (AML) being among the most aggressive and therapeutically challenging hematological malignancies (Wachter & Pikman 2024). AML is characterized by the clonal proliferation of abnormal myeloid precursors, leading to impaired hematopoiesis and rapid clinical deterioration if untreated (Vakiti et al. 2024; Wachter & Pikman 2024). The incidence is particularly high among individuals over 60 years old and is compounded by its genetically heterogeneous nature, which complicates treatment and contributes to variable prognoses (Vakiti et al. 2024; Wachter & Pikman 2024). Despite advances in targeted therapies, chemotherapy, and hematopoietic stem cell transplantation, many AML patients suffer from relapse, drug resistance, and treatment-related toxicity (Abaza et al. 2024; Bhagwat et al. 2024). These challenges underscore the urgent need for novel, more selective, and less toxic therapeutic agents. Over the last decade, rational drug design has emerged as a viable strategy for identifying inhibitors of dysregulated pathways involved in tumor progression (Yip & Papa 2021). Among these, protein kinases have garnered significant attention as druggable targets due to their regulatory roles in cell cycle progression, apoptosis, and proliferation (Bhullar et al. 2018).

However, the clinical utility of existing kinase inhibitors, such as gilteritinib and sorafenib, is often hampered by resistance development and adverse off-target effects (Shyam Sunder et al. 2023). Consequently, efforts have turned toward exploring novel scaffolds with better specificity and pharmacological profiles. Heterocyclic compounds, particularly those containing fused nitrogen rings, have shown immense therapeutic potential owing to their structural versatility and broad bioactivity spectrum (Bhullar et al. 2018). In this context, 1,2,4-triazolo[4,3-b]pyridazine derivatives have emerged as promising candidates. These tricyclic heterocycles possess physicochemical properties conducive to effective protein binding and have demonstrated significant anti-cancer activities in vitro (Pathan et al. 2023). Prior experimental studies confirmed that several of these derivatives, including compound 8l, exhibited potent antiproliferative activity against MV4-11 leukemia cells, with an IC₅₀ of 1.5 µM, and showed activity across multiple cancer cell lines (Pathan et al. 2023).

Given the relevance of Myeloid Cell Leukemia-1 (Mcl-1), a pro-survival BCL-2 family protein in AML pathogenesis and drug resistance, it represents an attractive target for therapeutic intervention (Nix & Price 2019; Tanaka et al. 2013). Several Mcl-1 inhibitors have been reported, yet issues related to selectivity and pharmacokinetics remain unresolved (Tanaka et al. 2013). In the current study, the focus on AML is driven by both the overexpression of Mcl-1 in AML and the prior demonstration of potent anti-AML activity of compound 8l (IC₅₀ = 1.5 µM)(Pathan et al. 2023). This association between Mcl-1 dysregulation and AML resistance justifies the specific disease focus and enables mechanistic investigation through targeted modeling.

Our integrated computational approach aligns with previous multi-tiered in silico strategies reported by Guendouzi and colleagues, who demonstrated the utility of combining molecular modeling with ADMET and quantum chemical analyses to optimize anti-cancer scaffolds (Guendouzi et al. 2024a, b, c). In particular, multiple studies have successfully applied molecular docking and molecular dynamics to explore Mcl-1 inhibition, validating the utility of computational pipelines for identifying and optimizing selective ligands for this protein (Alzain et al. 2025; Yue et al. 2023). While previous studies have successfully applied multi-tiered in silico methods to optimize anti-cancer scaffolds, our study is distinct in its focused application to Mcl-1 inhibition in the context of AML, leveraging a series of 1,2,4-triazolo[4,3-b]pyridazine derivatives. To our knowledge, this scaffold has not been comprehensively investigated for Mcl-1 targeting using an integrated computational pipeline that includes docking, MM/GBSA, MD simulations, frontier orbital analysis, and pharmacokinetic profiling. Moreover, while most existing in silico Mcl-1 inhibitor studies emphasize general anti-cancer utility, our work uniquely bridges prior experimental cytotoxicity data with structure-based modeling focused on AML. In this malignancy, Mcl-1 plays a pivotal survival role. This targeted approach not only enriches mechanistic understanding but also provides a rational scaffold for further optimization. While several BCL-2 family proteins, such as BCL-2 and BCL-xL, are implicated in AML pathogenesis, Mcl-1 has emerged as a particularly compelling target due to its rapid turnover, unique structural dynamics, and its central role in conferring resistance to chemotherapeutics and apoptosis (Ramsey et al. 2018; Wang et al. 2021). Furthermore, clinical resistance to existing BCL-2 inhibitors like venetoclax has been mechanistically linked to compensatory Mcl-1 upregulation in AML cells (Döhner et al. 2022; Thijssen et al. 2021). Thus, our decision to focus on Mcl-1 stems not only from its overexpression but also from its functionally non-redundant role in AML survival and relapse. While the current study explores Mcl-1 targeting explicitly, future work may expand this framework to include additional BCL-2 proteins for broader therapeutic synergy. Given the central role of Mcl-1 in AML cell survival and drug resistance, it was selected as the sole molecular target to enable detailed interaction profiling with the studied compounds. Thus, this study aims to explore the Mcl-1 binding potential and pharmacological viability of selected 1,2,4-triazolo[4,3-b]pyridazine derivatives through a robust computational framework, offering both mechanistic insights and a basis for future therapeutic development against AML.

Furthermore, this study builds upon earlier in vitro findings by Pathan et al. (2023), which demonstrated compound **8l** cytotoxicity against MV4-11 AML cells. In continuation of this work, our computational predictions will be subjected to experimental validation to bridge in silico modeling with translational pharmacology. Sunitinib was selected as a reference compound based on its inclusion in prior experimental studies involving 1,2,4-triazolo[4,3-b]pyridazine derivatives (Pathan et al. 2023), which reported potent anti-cancer activity, particularly against AML cell lines, maintaining consistency with this experimental benchmark allowed for a more meaningful comparison between in silico and in vitro results. While Sunitinib is not a selective Mcl-1 inhibitor, its broad-spectrum kinase inhibitory profile, established anti-leukemic activity (Oluwamodupe et al. 2025), and well-documented pharmacological properties make it a suitable standard for evaluating the binding affinity, stability, and pharmacokinetics of the designed compounds. Its use further supports continuity between synthetic efforts and computational optimization.

## Materials and methods

### Ligand, protein preparation, and receptor grid generation

The Schrödinger suite (Schrödinger, 2023–2) was selected for its well-established accuracy in molecular docking and free energy predictions, particularly for kinase and anti-cancer targets. Glide XP docking was employed for its enhanced scoring function, while MM/GBSA in Prime offered a reliable estimation of binding thermodynamics. MD simulations were run with Desmond using the OPLS4 force field (Roos et al. 2019) and TIP4P water model (Jorgensen et al. 1983) to reflect physiological solvation dynamics. Visualization and orbital analysis were conducted using GaussView 6 and Gaussian16, which were selected for their support of high-level DFT methods (Frisch & Schlegel 2016; Obakachi et al. 2025b). A total of twenty-six previously synthesized 1,2,4-triazolo[4,3-b]pyridazine derivatives were selected based on their reported anti-cancer activity (Pathan et al. 2023). Alongside the reference compound sunitinib, their chemical structures were sketched using and exported in Structure-Data File (SDF) format (ChemDraw 2018). Ligand preparation was carried out using LigPrep(Schrödinger, 2023–2), which involved energy minimization, salt neutralization, addition of hydrogen atoms, generation of valid stereoisomers, and ionization state prediction at physiological pH (7.0 ± 2.0). The geometries were optimized using the OPLS4 force field (Roos et al. 2019) to ensure low-energy conformations (Obakachi et al. 2025b).

The three-dimensional X-ray crystal structure of Myeloid Cell Leukemia-1 (Mcl-1) was obtained from the Protein Data Bank (PDB ID: 3WIX) (Tanaka et al. 2013), selected over other structures such as 6FSE and 6NE5 due to its favorable resolution, R-values, and superior docking performance in preliminary studies (Berman et al. 2002). Protein preparation was conducted using the Protein Preparation Wizard in Maestro (Schrödinger), which involved correcting bond orders, adding missing hydrogen atoms and side chains, assigning protonation states at pH 7.0, and removing water molecules beyond 5 Å from the active site. The structure was further energy-minimized using the OPLS4 force field to resolve steric clashes and ensure structural integrity.

**Fig. 1:**
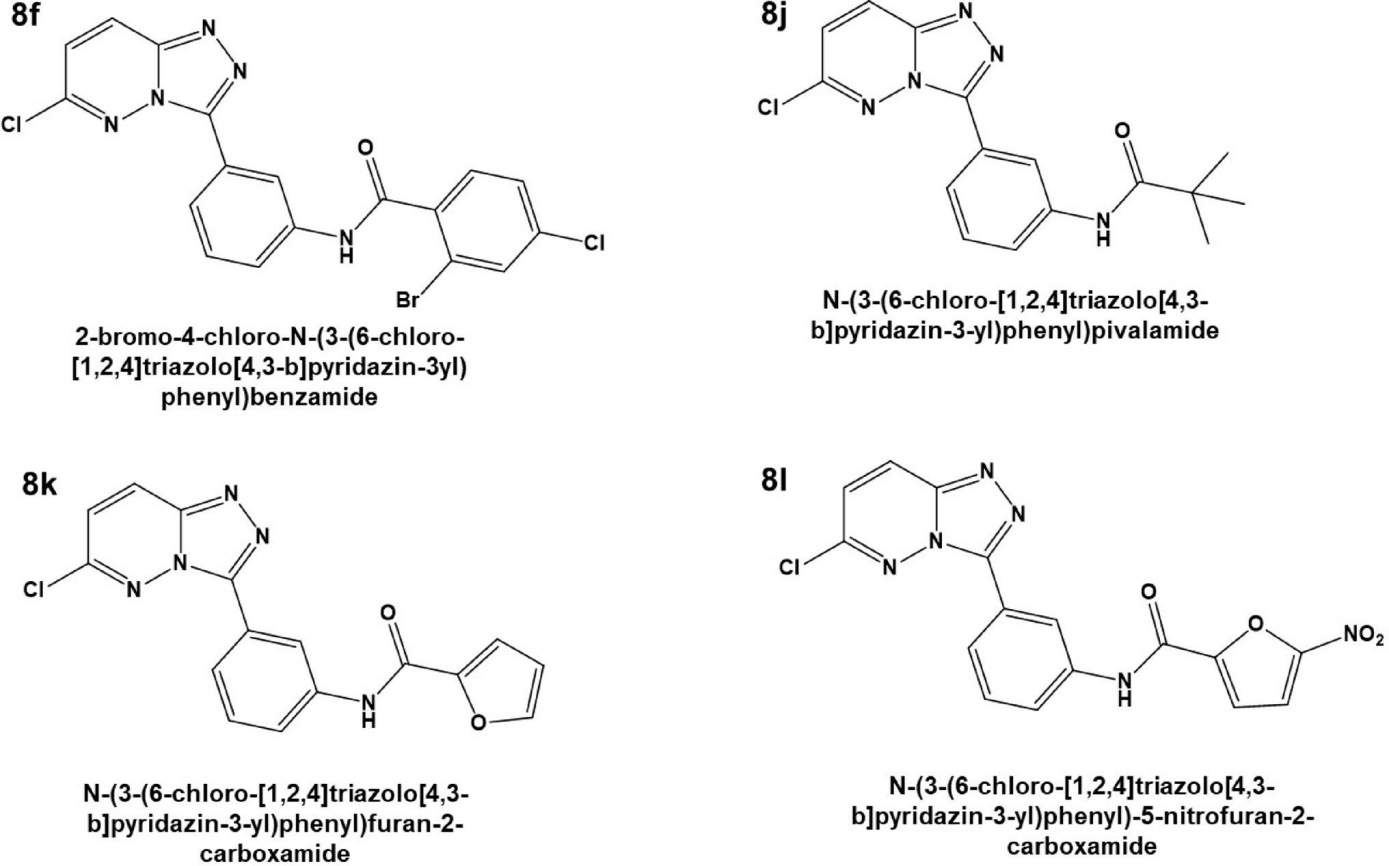
Two-Dimensional Schematic Representation of Lead Derivatives after Docking

Receptor grid generation was performed using the Glide module’s receptor grid generation tool, targeting the active site defined by the co-crystallized ligand (Obakachi et al. 2025a). A cubic grid was centered at coordinates (− 10.53 Å, 7.38 Å, − 56.34 Å), with an inner box size of 10 × 10 × 10 Å and an outer box size of 27.41 × 27.41 × 27.41 Å. Each ligand was then docked into the grid using Glide Extra Precision (XP) mode, which offers enhanced accuracy over standard precision docking (Friesner et al. 2004). Binding poses were evaluated based on Glide XP docking scores, and the best-ranked conformations were retained for subsequent analyses. The 1,2,4-triazolo[4,3-b]pyridazine derivatives used in this study were previously synthesized and biologically evaluated by Pathan et al. (2023). No new chemical synthesis was performed in this work; rather, the current study focused on computational evaluation of the reported structures (Fig. 1) (Pathan et al. 2023).

### Protein structure validation using ramachandran plot

**Fig. 2:**
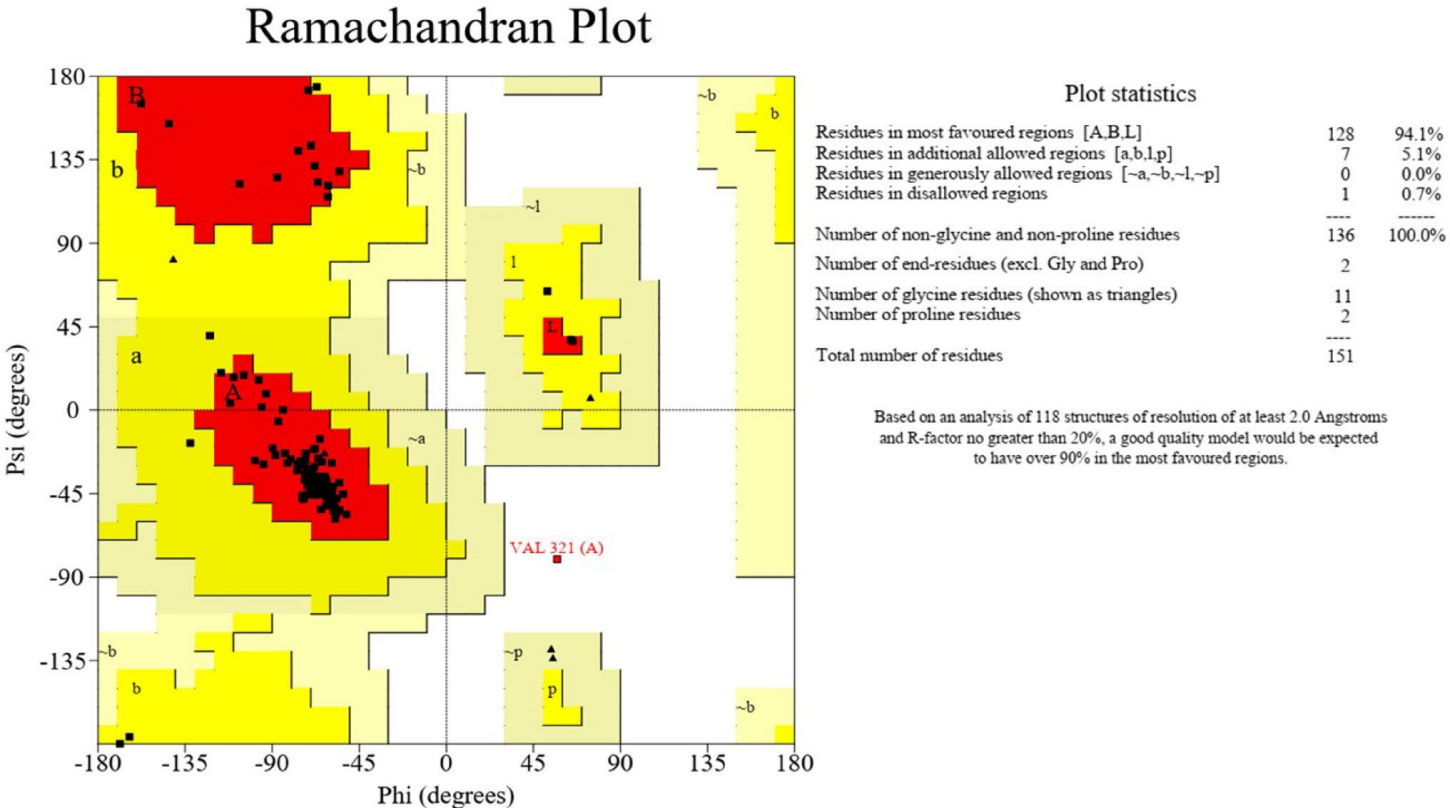
Ramachandran plot of the Mcl-1 protein structure generated using PROCHECK

To ensure the stereochemical reliability of the protein model (PDB ID: 3WIX) used in this study, structural validation was performed using PROCHECK (Laskowski et al. 1993). The Ramachandran plot generated (Fig. 2) highlights the distribution of backbone dihedral angles (φ, ψ) for all residues in the protein. Over 98% of residues were located in the most favorable and additionally allowed regions, confirming the high structural integrity of the protein. This validation supports the suitability of the model for subsequent molecular docking and simulation studies.

### Docking simulation

The crystal structure of Mcl-1 (PDB ID: 3WIX) was selected for molecular docking based on its high resolution (1.90 Å), completeness (4054 atoms), and relevance to inhibitor binding. This structure contains a co-crystallized pyrazolo[1,5-a]pyridine-based ligand (LC3) occupying the canonical BH3-binding groove, which is critical for targeting anti-apoptotic Mcl-1 in cancer. The biologically relevant conformation of the binding site and its established use in previous Mcl-1 docking studies justified its suitability for our AML-focused analysis. Molecular docking studies were conducted using the Glide module in the Schrödinger Suite 2023–2 (Schrödinger, 2023–2). The receptor (Mcl-1) was treated as a rigid body, while ligand flexibility was preserved throughout the docking process. A docking grid was generated around the co-crystallized ligand (LC3), with dimensions optimized to encompass the active site. Default van der Waals scaling factors (0.80) and a partial charge cutoff of 0.15 were employed during receptor grid generation, consistent with best practices for accuracy and computational efficiency. Docking was performed in Extra Precision (XP) mode to enhance binding pose discrimination and scoring reliability. The scoring function incorporated terms for binding affinity, internal energy, and desolvation, facilitating identification of the most favorable ligand-receptor interactions. Post-docking analysis was conducted using Glide XP Visualizer, focusing on key interactions such as hydrogen bonds, π-π stacking, hydrophobic contacts, and electrostatic contributions. These interaction profiles were further evaluated to prioritize ligand candidates for downstream analysis.

### Inhibition constant (Ki) calculation

The inhibition constant (Ki) (Yung-Chi & Prusoff 1973) values for the docked complexes were estimated from the Glide docking scores using the thermodynamic relationship:

$$ {\text{Ki}} = {\text{ e}}^{{\Delta {\text{G}} \times {1}000/{\text{RT}}}} $$

where:

ΔG = binding affinity (in kcal/mol).

R = 1.987 cal/mol·K (gas constant).

T = 298.15 K (room temperature).

Ki is in Molar (M) ( then converted to micromolar (µM) by multiplying by 10^6^).

The ΔG values were obtained directly from the Glide docking scores, and the resulting Ki values provide a comparative estimate of binding affinity in molar units. These values were included in Table 1 to complement the GlideScore data.

### Validation of docking protocol

**Fig. 3:**
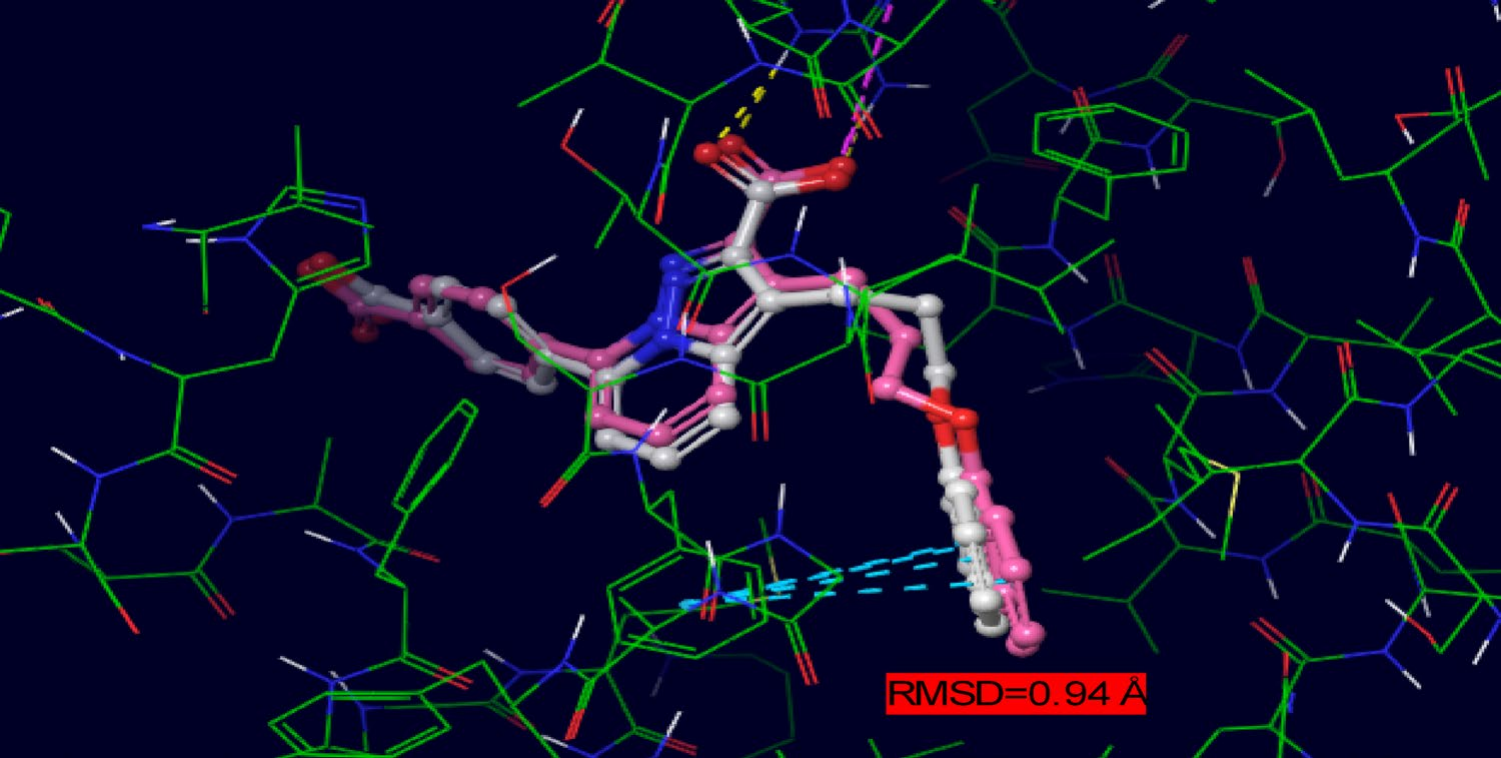
Structural Alignment of LC3 (white ball and stick) with the Native pose of co-crystallized LC3 (pink ball and stick) extracted from the PDB in the protein’s binding site

To ensure the reliability of the docking methodology, the co-crystallized ligand LC3 was removed from the Mcl-1 binding site (PDB ID: 3WIX) and re-docked using the same docking protocol (Glide XP mode) without applying any positional constraints. The re-docked conformation exhibited a strong structural overlap with the native pose, yielding a root mean square deviation (RMSD) of 0.94 Å, which is well within the acceptable threshold for validation of docking accuracy (Repasky et al. 2007). This result confirms that the employed docking protocol can accurately reproduce experimentally observed binding orientations and interactions within the Mcl-1 active site. Following successful validation, the prepared receptor grid was retained, and all synthesized 1,2,4-triazolo[4,3-b]pyridazine derivatives were docked under the same conditions. Molecular interaction maps were generated to examine key contacts within the active site. Nonpolar hydrogens and less relevant amino acid residues were omitted in the visual representations for clarity (Fig. 3). The docking outcomes served as the basis for selecting the most promising candidates for further dynamic and energetic evaluation.

### Molecular dynamics simulation protocol

Molecular dynamics (MD) simulations were performed using the Desmond module in the Schrödinger Suite (Bowers et al. 2006) to investigate the structural stability and dynamic behavior of the top-scoring protein–ligand complexes (Schrödinger Release 2023–2: Desmond Molecular Dynamics System). Complexes were selected based on their Glide XP docking scores and key molecular interactions observed during post-docking analysis. Each complex was prepared using Desmond’s System Builder, where it was placed in a truncated octahedral simulation box under periodic boundary conditions. The systems were solvated using the TIP4P explicit water model(Jorgensen et al. 1983), ensuring a minimum buffer distance of 10 Å between the solute and the box boundaries. To mimic physiological ionic strength, Na⁺ and Cl⁻ counterions were added to neutralize the system, along with 0.15 M NaCl to simulate physiological saline conditions. The OPLS4 force field was used to model both bonded and non-bonded interactions within the protein–ligand solvent system (Huang et al. 2005). After solvation, energy minimization was carried out using a steepest descent algorithm with a convergence threshold of 1.0 Å to relieve steric clashes and optimize geometry. A two-stage equilibration protocol was followed: first, a 1 ns equilibration in the NVT ensemble (constant number of particles, volume, and temperature) at 300 K, and then a 1 ns NPT ensemble run at 300 K and 1 atm to stabilize system pressure and density (Berendsen et al. 1984). Subsequently, a 200 ns production MD simulation was conducted under NPT conditions, with a 2 fs time step. Atomic coordinates, system energies, and trajectory data were recorded every 10 ps for downstream analysis of stability metrics such as RMSD, radius of gyration, RMSF, and hydrogen bond persistence.

### Post-MD simulation analysis

To comprehensively assess the stability and behavior of the protein–ligand complexes during molecular dynamics (MD) simulations, several post-simulation analyses were performed. Key metrics included root mean square deviation (RMSD), root mean square fluctuation (RMSF), and binding interaction profiles. These evaluations were conducted using the Simulation Interaction Diagram (SID) tool in Desmond (Schrödinger Release 2023–2: Desmond Simulation Interaction Diagram Tool). The RMSD plots provided insight into the structural convergence and dynamic equilibrium of the complexes. A plateau phase in the RMSD trajectory was indicative of system stability and was used as a criterion to select stable segments for downstream analysis (Phillips et al. 2005). RMSF plots were also generated to quantify the flexibility of individual residues and to identify dynamically stable regions within the protein backbone during ligand binding. To quantify binding affinity, binding free energy (ΔG_bind_) calculations were performed using the MM/GBSA (Molecular Mechanics/Generalized Born Surface Area) method, implemented via the Prime MM-GBSA module(Lyne et al. 2006). Specific trajectory frames corresponding to stable RMSD regions were selected to ensure statistically robust energy calculations. Prior to analysis, solvent molecules and counterions were excluded to focus exclusively on protein–ligand interactions. The MM/GBSA protocol involved decomposition of the binding energy into key thermodynamic and interaction components, including:

ΔG_coulomb_—Coulombic interaction energy.

ΔG_covalent_—Covalent bonding contributions.

ΔG_Hbond_—Hydrogen bonding corrections.

ΔG_vdw_—van der Waals interactions.

ΔG_Lipo_—Nonpolar (lipophilic) solvation.

ΔG_packing_—π-π stacking correction.

ΔG_solv_—Generalized Born electrostatic solvation energy.

The total binding free energy, ΔG_bind,_ was calculated according to Eq. (1):

1 $$\begin{aligned} \Delta {\text{G}}_{{{\text{bind}}}} = & \Delta {\text{G}}_{{{\text{coulomb}}}} + {\mkern 1mu} \Delta {\text{G}}_{{{\text{covalent}}}} + {\mkern 1mu} \Delta {\text{G}}_{{{\text{Hbond}}}} \\ & + {\mkern 1mu} \Delta {\text{G}}_{{{\text{vdw}}}} + {\mkern 1mu} \Delta {\text{G}}_{{{\text{Lipo}}}} + {\mkern 1mu} \Delta {\text{G}}_{{{\text{packing}}}} + {\mkern 1mu} \Delta {\text{G}}_{{{\text{solv}}}} \\ \end{aligned}$$

The job was executed through a terminal interface using a bash script, and the resulting data were exported in CSV format for visualization and further interpretation. These results provided a detailed breakdown of interaction energy contributions that govern ligand binding affinity and complex stability.

### Density functional theory (DFT)

The electronic properties and chemical reactivity of the top hit compounds were investigated using Density Functional Theory (DFT) (Frisch & Schlegel 2016; Obakachi et al. 2025a; Vijayaraj et al. 2009) to gain deeper insight into their interaction potential and stability. Specifically, the frontier molecular orbitals, the highest occupied molecular orbital (HOMO) and lowest unoccupied molecular orbital (LUMO), were analyzed to estimate the compounds’ ability to donate or accept electrons. The HOMO–LUMO energy gap (ΔE) served as a key descriptor of electronic reactivity and kinetic stability. Electrostatic Potential (ESP) maps were generated to visualize charge distribution and identify regions susceptible to nucleophilic or electrophilic attack (Lu & Chen 2012). Prior to this, all ligand geometries were fully optimized using the B3LYP hybrid functional in conjunction with the DEF2TZVP (Weigend & Ahlrichs 2005) basis set as implemented in Gaussian 16 software (Frisch & Schlegel 2016). Vibrational frequency analyses were performed to confirm the reliability of the optimized structures. The absence of imaginary frequencies validated that the structures correspond to local minima on the potential energy surface. This step also supported the evaluation of Molecular Electrostatic Potential (MEP) surfaces and the accurate estimation of reactivity descriptors. Following geometry optimization, single-point energy calculations were performed both in the gas phase and in dimethylformamide (DMF), the experimental solvent used in previous in vitro studies. Solvation effects were modeled using the Integral Equation Formalism Polarizable Continuum Model (IEFPCM) to simulate realistic environmental conditions (Tomasi et al. 2005). All DFT calculations were executed using Gaussian 16 on the Lengau cluster at the Centre for High-Performance Computing (CHPC) in Cape Town, South Africa. This protocol allowed for a detailed theoretical assessment of each compound’s electronic behavior under both ideal and solvent-influenced conditions. All quantum chemical calculations were performed using Gaussian 16. Visualization and analysis of frontier molecular orbitals (FMO) and molecular electrostatic potential (MEP) maps were conducted using GaussView 6. The solvent effects were modeled using the Integral Equation Formalism Polarizable Continuum Model (IEFPCM) to account for bulk solvation behavior (Tomasi et al. 2005), with the dielectric constant (ε) set to 36.7, corresponding to dimethylformamide (DMF).

### ADME-tox properties

The absorption, distribution, metabolism, excretion, and toxicity (ADME-Tox) profiles of the selected compounds were evaluated to assess their pharmacokinetic suitability and drug-likeness. These analyses were conducted using the QikProp module within the Maestro Schrödinger Suite (Schrödinger Release 2023–2: QikProp). QikProp provides a robust in silico platform for predicting key physicochemical and pharmacokinetic properties, including solubility, permeability, oral absorption, and blood–brain barrier penetration, thereby aiding in the early-stage assessment of drug candidates. In addition, QikProp evaluates each compound’s compliance with Lipinski’s Rule of Five, a widely recognized benchmark for predicting oral bioavailability. According to this rule, a compound is more likely to exhibit good oral absorption if it meets the following criteria: (i) no more than five hydrogen bond donors, (ii) no more than ten hydrogen bond acceptors, (iii) molecular weight less than 500 Da, and (iv) an octanol–water partition coefficient (log P) less than five (Lipinski et al. 1997). These computed parameters are essential for identifying compounds with favorable drug-like properties, enabling the selection of candidates with optimized absorption and bioavailability profiles. The insights gained from QikProp streamline decision-making in lead optimization by highlighting molecules with optimal pharmacokinetic behavior and minimal violation of drug-likeness rules. Furthermore, toxicity predictions were performed using the ProTox-II webserver (version 3.0), which employs machine learning and molecular similarity approaches to estimate toxicity endpoints such as LD_50_, hepatotoxicity, carcinogenicity, mutagenicity, and immunotoxicity (Banerjee et al. 2018). The combined ADME-Tox profiling thus offers a comprehensive assessment of the therapeutic potential and safety margins of the screened compounds prior to preclinical testing.

### Planned experimental validation

To support the computational findings, we are initiating a series of wet-lab validation studies with our collaborators. Compound **8l** and other optimized analogs will be evaluated for Mcl-1 inhibition using surface plasmon resonance (SPR) and enzyme inhibition assays with purified protein. Additionally, in vitro antiproliferative activity will be assessed using MTT assays on MV4-11 and HL-60 AML cell lines. These experimental approaches aim to validate the predicted binding interactions and confirm the biological efficacy of the lead compound.

## Results and discussion

### Protein structure validation

To ensure the structural quality of the Mcl-1 protein used in docking and simulations, a Ramachandran plot analysis was performed using PROCHECK (Fig. 2). The results showed that 94.1% of the residues fell within the most favored regions (A, B, L), and an additional 5.1% were located in allowed regions (a, b, l, p). Only 0.7% of residues were found in disallowed regions, well within the acceptable range for a high-quality model. These statistics confirm the stereochemical integrity and reliability of the protein structure used in this study, supporting the validity of subsequent docking and simulation analyses (Laskowski et al. 1993).

### Molecular docking and antiproliferative evaluation

A comprehensive computational docking study was carried out to evaluate the binding interactions between the synthesized 1,2,4-triazolo[4,3-b]pyridazine derivatives and Myeloid Cell Leukemia-1 (Mcl-1), a critical anti-apoptotic protein implicated in Acute Myeloid Leukemia (AML) pathogenesis (Tanaka et al. 2013). The objective was to identify lead candidates with high binding affinity and experimentally validated antiproliferative activity against MV4-11 cells, an established AML cell line model. The crystal structure of Mcl-1 (PDB ID: 3WIX) was retrieved from the Protein Data Bank (Berman et al. 2002) and used as the receptor in docking simulations, which were executed using the Glide module within the Schrödinger Suite (Schrödinger, 2023–2). Docking was performed in Extra Precision (XP) mode, which provides robust predictions of ligand–protein binding poses and binding affinity. The docking analysis focused on 26 synthesized derivatives, assessing their ability to interact with the Mcl-1 active site. Key docking metrics included the Glide Score (binding affinity), Glide Energy (interaction energy), and Glide Emodel (empirical energy model incorporating pose quality). The results for the top-performing compounds **8f**, **8j**, **8k**, **8l**, and Sunitinib are summarized in Table 1.

**Table 1:** Glide docking scores, estimated inhibition constants (Ki), and antiproliferative activity (IC₅₀) of the tested compounds against MV4-11 leukemia cells

Compd name	IC_50_ µM MV4-11	Glide Score (kcal/mol)	Glide emodel (kcal/mol	Glide energy (kcal/mol)	Estimated Ki (µM)
8f	20.70 ± 4.80	− 8.91	− 73.71	− 47.85	− 0.31
8j	21.50 ± 2.70	− 8.85	− 60.58	− 46.08	− 0.32
8k	4.70 ± 1.30	− 8.75	− 68.02	− 47.24	− 0.35
8l	1.50 ± 0.50	− 8.81	− 72.02	− 48.90	− 0.34
Sunitinib	0.05 ± 0.04	− 8.74	− 56.73	− 44.48	− 0.36

As illustrated in Fig. 1, compounds **8f**, **8j**, **8k**, and **8l** displayed more favorable Glide scores than the reference compound sunitinib (− 8.74 kcal/mol) (Costanzo et al. 2008), suggesting superior predicted binding affinities to Mcl-1. Among these, compound **8l** stood out with a Glide Score of − 8.81 kcal/mol and the lowest Glide Energy (− 48.90 kcal/mol), indicative of strong and stable protein–ligand interactions. These computational findings are consistent with previous experimental results, which reported potent antiproliferative activity of compound **8l** against MV4-11 cells (IC₅₀ = 1.5 ± 0.5 µM) (Pathan et al. 2023).

In contrast, compounds with lower Glide scores (− 8.49 to − 3.28 kcal/mol) displayed comparatively weaker predicted affinities (Supplementary Material S1). While Glide scoring provides an effective initial filter for identifying promising binders, it does not fully account for the dynamic flexibility of the protein–ligand complex under physiological conditions (J. C. Smith, 2014). Therefore, although compound **8l** demonstrated superior static docking metrics, further validation was pursued via molecular dynamics (MD) simulations, which offer a more realistic assessment of binding stability and conformational adaptability. The integration of docking scores, empirical binding energies, and previous experimental data validated compound **8l** as a compelling lead candidate for further development. Compounds **8f**, **8j**, and **8k** also showed favorable docking and biological profiles, warranting extended computational and experimental evaluation. Additionally, inhibition constants (Ki) derived from docking scores were estimated to further assess binding potency(Table 1). Compounds **8f**, **8j**, **8k**, and **8l** yielded Ki values of 0.31 µM, 0.32 µM, 0.35 µM, and 0.34 µM, respectively. Sunitinib displayed a slightly weaker Ki of 0.36 µM, in agreement with its lower Glide score. These values reinforce compound **8l** potency, despite its moderate solubility and permeability limitations, supporting its potential as a lead scaffold for Mcl-1 inhibition. Subsequent MD simulations were employed to evaluate the temporal stability and interaction persistence of these top-ranking ligands within the Mcl-1 binding pocket. This combination of static (docking) and dynamic (MD) analyses ensured a comprehensive understanding of the therapeutic potential of the selected candidates under near-physiological conditions (Hospital et al. 2015).

### Protein–ligand(P-L) interaction analysis

**Fig. 4:**
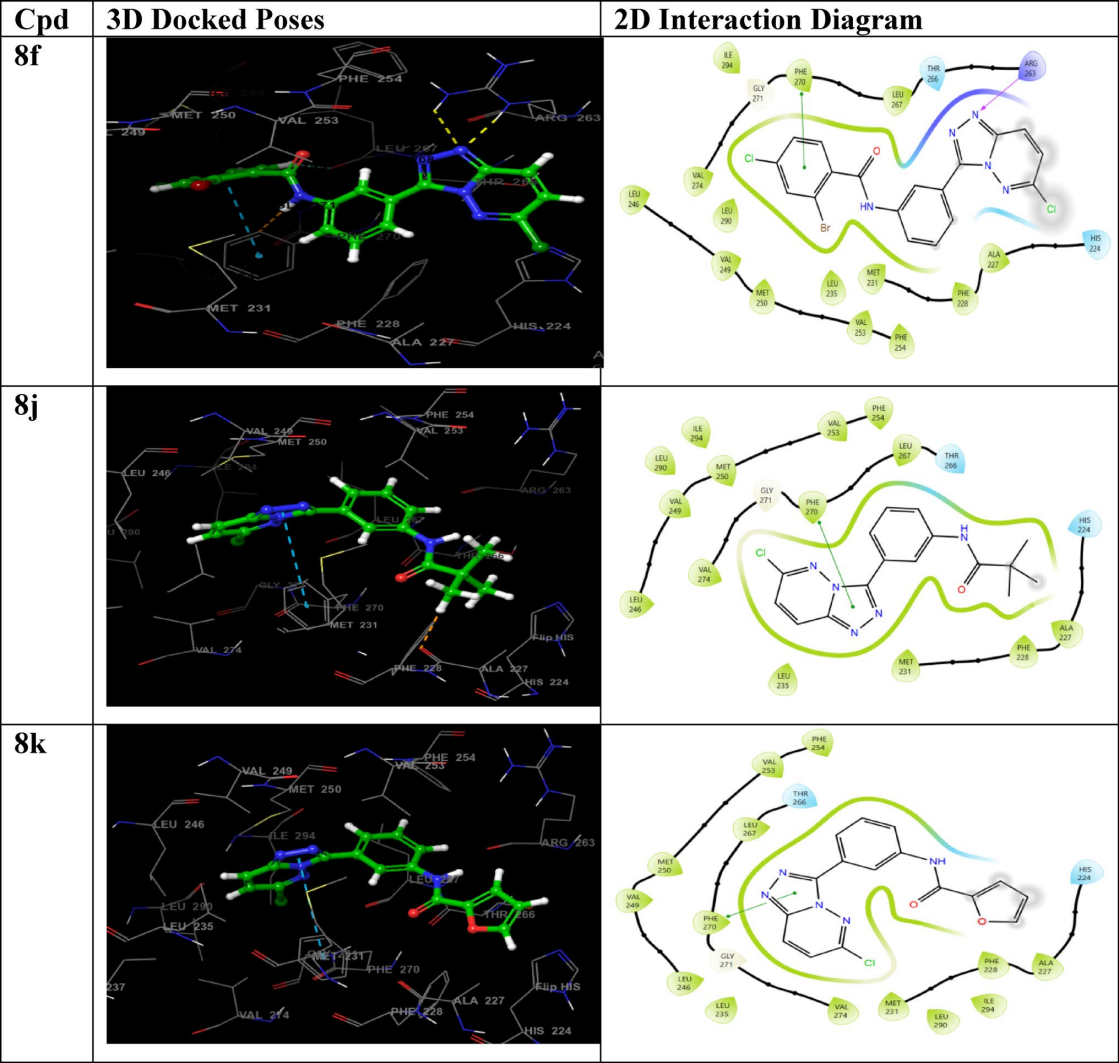

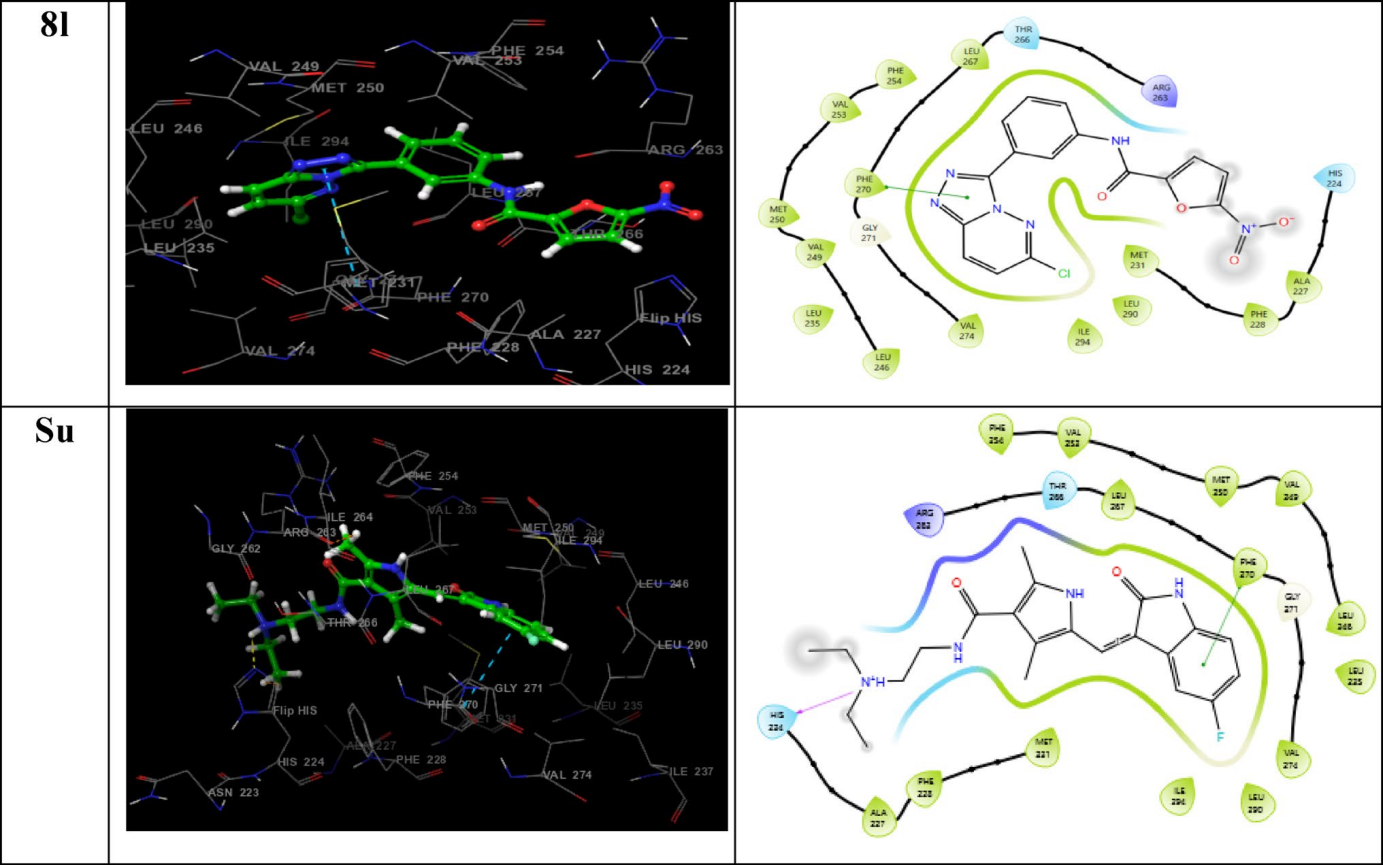
3D and 2D docked poses and protein–ligand interactions of lead molecules and reference

The P-L interaction, as shown in Fig. 4, illustrates the binding interactions of lead compounds **8j**, **8k**, **8l**, and **8f** within the active site of the Mcl-1 receptor, highlighting critical hydrophobic and polar contacts with key residues such as LEU267, MET231, VAL274, PHE228, and ALA227. Notably, compounds **8j**, **8k**, and **8l** exhibited a shared interaction profile, forming π-π stacking interactions between their triazole-pyridazine scaffold and PHE270, a residue known to participate in stabilizing ligand binding within the Mcl-1 active site (Bolomsky et al. 2020).

In contrast, compound **8f** displayed a distinct binding mode, establishing a π-π stacking interaction via the phenyl ring of its benzamide moiety, diverging from the triazole-mediated binding observed in the other derivatives. Additional polar contacts with residues such as THR266 and HIS224 further contributed to the stabilization of the protein–ligand complexes, enhancing binding affinity. The reference compound sunitinib also demonstrated a comparable interaction pattern to **8j**, **8k**, and **8l**, engaging PHE270 through its fused triazole-pyridazine system. However, the subtle differences in interaction profiles, especially that of **8f,** underscore the influence of peripheral substituents on binding orientation and strength. Collectively, these interaction patterns support the strong binding potential of the lead compounds and reinforce their viability as Mcl-1 inhibitors in the context of AML therapy (Friesner et al. 2004).

### Molecular dynamics (MD) simulation analysis

Molecular dynamics (MD) simulations were performed to investigate the time-dependent behavior, relaxation, and conformational stability of Mcl-1 protein complexes with the most promising ligands. Simulations were carried out for 200 ns using the Desmond module in Schrödinger (Schrödinger Release 2023–2: Desmond Molecular Dynamics System), employing the docked poses of ligand–protein complexes as starting configurations. MD simulations are a well-established approach for assessing the physical realism of protein–ligand binding and provide insights beyond static docking by revealing how molecular interactions evolve under near-physiological conditions (Obakachi et al. 2021).

**Fig. 5:**
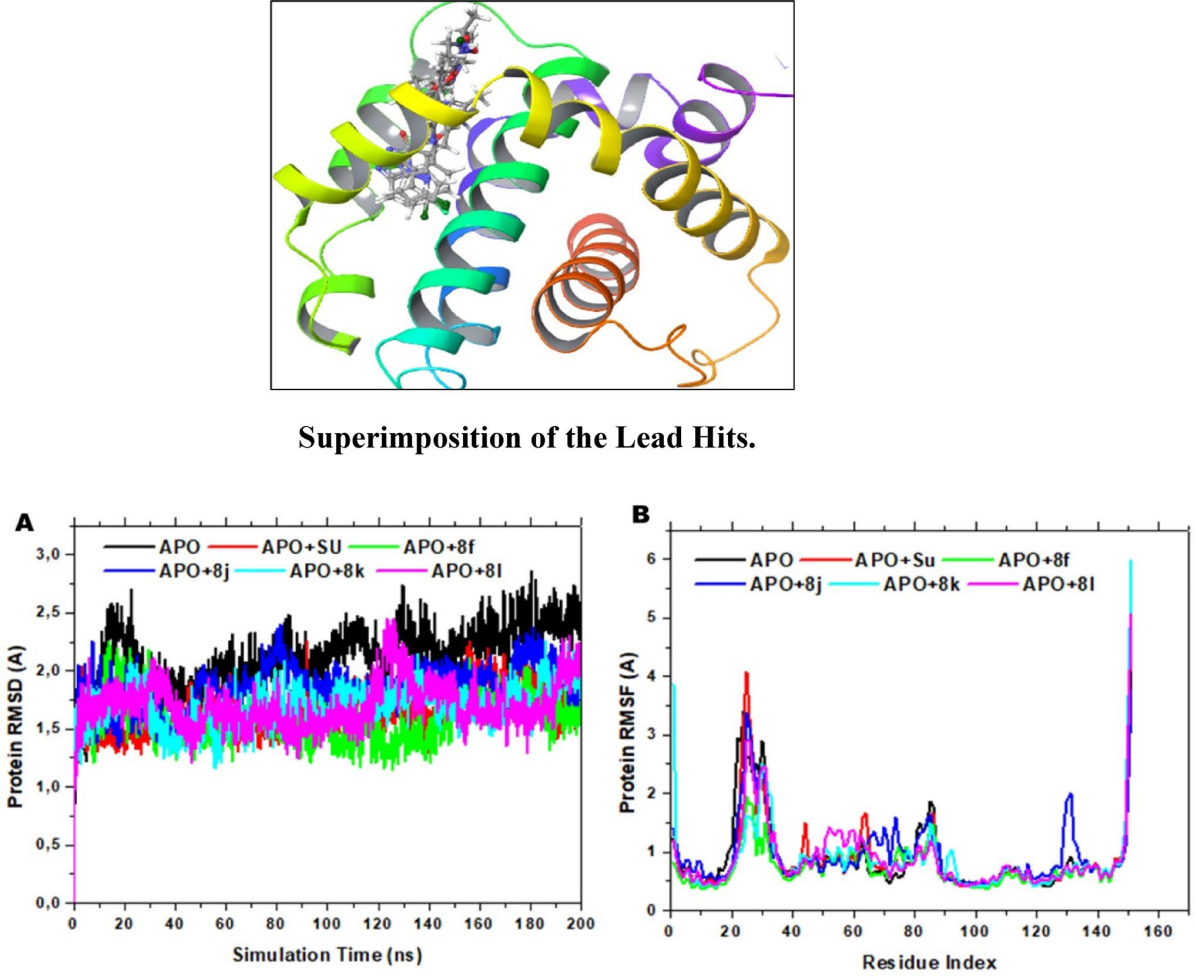
**A** RMSD and** B** RMSF plots over 200 ns MD simulations showing improved Mcl-1 stability and reduced flexibility upon ligand binding compared to the APO form

Key metrics, including root mean square deviation (RMSD) and root mean square fluctuation (RMSF) of the protein backbone, were analyzed to assess system stability(Ibrahim et al. 2025). As shown in Fig. 5, all protein–ligand complexes achieved structural equilibration following an initial phase of relaxation, with relatively low fluctuations maintained throughout the 200 ns simulation. The unbound (APO) structure displayed an average RMSD of 2.12 Å, indicating a moderate degree of structural flexibility in the absence of ligand binding. Upon ligand binding, the protein RMSD values decreased, reflecting increased stabilization. Specifically, the **8f** complex exhibited the lowest average RMSD of 1.68 Å, followed closely by **8l** at 1.71 Å, while **8k** and Sunitinib (Su) had average RMSD values of 1.76 Å and 1.88 Å, respectively. These results suggest that compounds **8f** and **8l** effectively stabilized the Mcl-1 structure, comparable to or exceeding the performance of the reference compound.

The RMSF analysis (Fig. 4) provided further insight into the local flexibility of amino acid residues(Shaik et al. 2025). The APO structure showed an average RMSF of 0.92 Å, establishing a baseline for comparison. Ligand binding generally reduced the fluctuation of key residues, indicating constrained conformational mobility. Among the tested systems, the **8f** complex showed the most pronounced reduction in flexibility, with an average RMSF of 0.76 Å, suggesting significant stabilizing effects on the protein structure. This was followed by **8k** (0.87 Å) and **8l** (0.89 Å), which showed comparable stabilization to the Su complex (0.89 Å). On the other hand, the **8j** complex exhibited the highest RMSF value of 0.96 Å, indicating increased flexibility and weaker stabilization. In all, the MD simulation results highlight the stabilizing influence of ligands **8f**, **8k**, and **8l** on the Mcl-1 receptor. Compound **8f** induced the most structural rigidity, while compound **8l** demonstrated consistent dynamic stability and reaffirmed its status as the top-performing compound, reaffirming its previously reported potent antiproliferative activity (IC₅₀ = 1.5 µM) against Mcl-1 in experimental studies. The low RMSD and RMSF values for compound **8l** suggest its stable and consistent interaction with Mcl-1, confirming its ability to maintain optimal binding orientation over the simulation timescale. These metrics provide dynamic support to static docking predictions, highlighting **8l** potential for sustained inhibitory action. In contrast, the slightly higher flexibility observed for **8j** may contribute to its relatively lower binding energy. Overall, the findings support the continued development of **8l** and **8f** as promising candidates for targeting Mcl-1 in AML therapy.

### Protein–ligand contact analysis

**Fig. 6:**
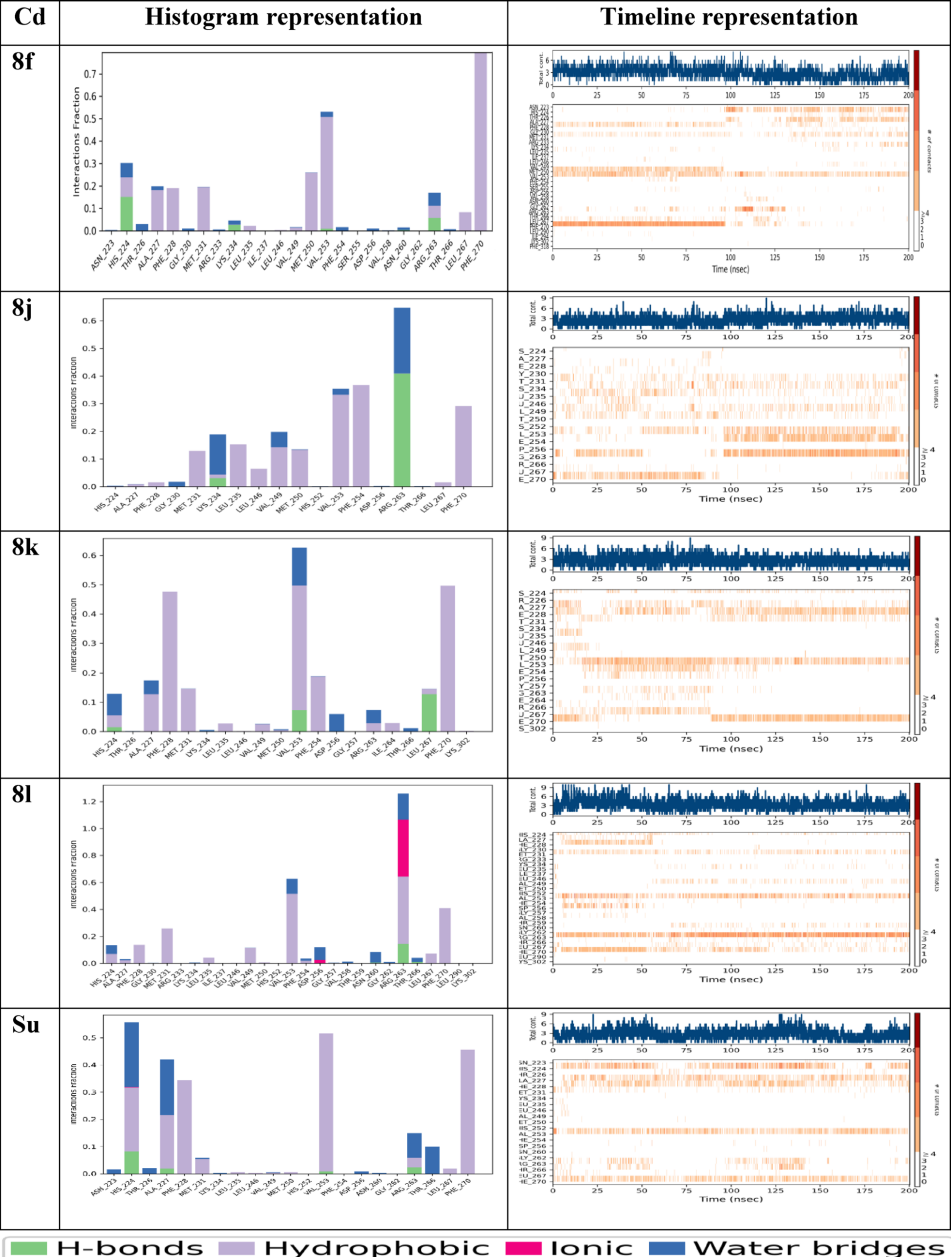
Showing the histogram representation of the count of the interaction and timeline representation of the interactions and contacts of 8f, 8j, 8k, 8l, and Su with the protein(3WIX)

As shown in Fig. 6, the interaction profiles of Mcl-1 with compounds **8f**, **8j**, **8k**, **8l**, and the reference sunitinib were monitored over 200 ns MD simulations and categorized into hydrogen bonds, hydrophobic contacts, ionic interactions, and water bridges. These dynamic contacts are key contributors to complex stabilization and reflect the ligands’ potential binding efficiency.

Across all systems, PHE270 consistently emerged as a critical residue, maintaining near-continuous hydrophobic or π-π stacking interactions, particularly with compounds **8f**, **8j**, **8k**, and **8l**. VAL253, MET231, MET250, and PHE254 also frequently contributed to hydrophobic stabilization, with VAL253 exhibiting long-duration contacts across all ligands. HIS224, observed in both **8f** and sunitinib complexes, engaged in hydrogen bonds and water bridges, contributing to complex stability, especially in the later simulation stages.

ARG263 played a significant role in stabilizing **8j**, **8l**, and Sunitinib through hydrogen bonding and ionic interactions. It maintained up to 40% interaction frequency with **8j** and contributed consistently to **8l’s** and Sunitinib’s stability via hydrogen bonds, water bridges, and electrostatic contacts. LEU267 and ALA227 were also involved in ligand-specific interactions, especially in the early phases of the trajectory. Among the lead compounds, **8l** demonstrated the most balanced and persistent interactions across all contact types, including significant contributions from ARG263, PHE270, and MET231, aligning with its superior antiproliferative potency (IC₅₀ = 1.5 µM). **8f** and **8 k** also showed stable and diverse interaction profiles, though **8j** displayed more transient and flexible binding, consistent with its relatively higher RMSD and lower experimental potency. Overall, the interaction landscape underscores the importance of PHE270, VAL253, ARG263, and HIS224 in anchoring ligands within the Mcl-1 binding site. These findings reinforce compound **8l** dynamic stability and strong binding potential, supporting its candidacy for further optimization. Our integrated computational approach aligns with previous multi-tiered in silico strategies reported by Guendouzi and colleagues, who demonstrated the utility of combining molecular modeling with ADMET and quantum chemical analyses to optimize anti-cancer scaffolds (Guendouzi et al. 2024a, b, c).

### Binding free energy (MM/GBSA) analysis

**Fig. 7:**
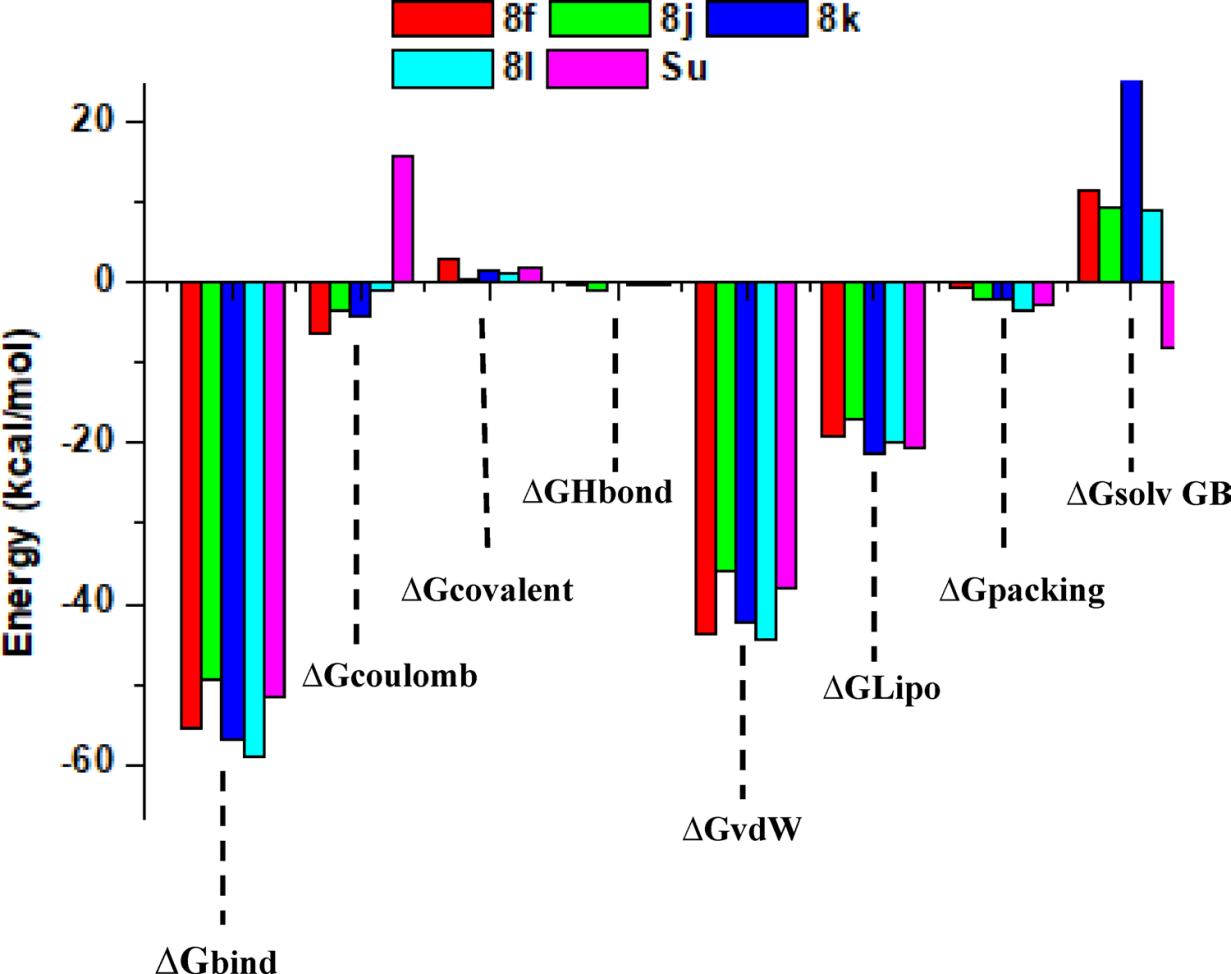
Illustrate the Binding Energy Contributions Pictorially

The MM/GBSA binding free energy (ΔG_bind_) values of the lead compounds (**8f**, **8j**, **8k**, **8l**) and the reference compound sunitinib (Su) were calculated to evaluate the thermodynamic stability of their interactions with Mcl-1. As summarized in Table 2 and illustrated in Fig. 7, all lead compounds demonstrated more favorable ΔG_bind_ values than Sunitinib, with compound **8l** showing the strongest binding affinity (− 58.96 kcal/mol), followed by **8k** (− 57.09 kcal/mol) and **8f** (− 55.51 kcal/mol). Sunitinib displayed a comparatively weaker ΔG_bind_ of − 51.71 kcal/mol, reinforcing the superior stability of the lead compounds.

**Table 2:** Binding Free Energy parameter profile of the Lead compounds

Parameters	8f	8j	8k	8l	Su
∆G_bind_	− 55.51	− 49.53	− 57.09	− 58.96	− 51.71
∆G_Coulomb_	− 6.28	− 3.47	− 4.30	− 1.11	15.70
∆G_Covalent_	3.00	0.63	1.37	1.07	1.99
∆G_Hbond_	− 0.29	− 1.03	− 0.02	− 0.07	− 0.21
∆G_vdW_	− 43.79	− 35.86	− 42.29	− 44.43	− 37.89
∆G_Lipo_	− 19,09	− 17.1	− 21.4	− 19.76	− 20.50
∆G_packing_	− 0.72	− 1.96	− 2.12	− 3.62	− 2.74
∆G_solv GB_	11.66	9.34	117.4	8.97	− 8.04

The energy decomposition, as shown in Table 2 and Fig. 7, revealed that van der Waals and lipophilic interactions were the dominant stabilizing forces across all ligands, particularly for **8l**, **8k**, and **8f**, while covalent and hydrogen bonding contributions remained modest. Electrostatic interactions varied considerably; **8f** contributed the most favorably in this category, whereas **8l** relied least on Coulombic stabilization. Interestingly, Sunitinib exhibited a positive Coulombic energy, indicative of repulsive electrostatic interactions. Packing efficiency was highest in compound **8l**, reflecting a superior steric fit within the binding pocket. The dominance of van der Waals and lipophilic contributions suggests that hydrophobic interactions are critical for stabilizing the ligand–protein complex. This emphasizes the importance of optimizing the hydrophobic moieties on the ligand scaffold. Additionally, the high packing efficiency of **8l** could explain its tight steric fit and enhanced thermodynamic stability, despite lower electrostatic contributions. Although solvation penalties were observed for all lead compounds, especially **8f** and **8k**, these were outweighed by strong nonpolar contributions. Sunitinib was the only compound to show a favorable negative solvation energy, though its overall binding energy remained less favorable. Collectively, these findings confirm that the lead compounds, particularly **8l** and **8k,** exhibit enhanced thermodynamic stability relative to Sunitinib, driven primarily by hydrophobic, dispersion, and steric interactions. These results suggest that compound **8l** engages in stronger binding interactions within the Mcl-1 pocket, with more favorable binding free energy compared to Sunitinib. The consistent presence of key non-covalent interactions such as hydrogen bonding and π-π stacking likely explains its enhanced affinity, aligning with experimental IC₅₀ values. The docking poses further demonstrate the importance of the [1,2,4]-triazolo[4,3-b]pyridazine scaffold in optimal binding orientation and previously reported biological potency.

### Quantum chemical calculations

To evaluate the electronic behavior and reactivity of the lead compounds, DFT-based quantum chemical calculations were performed in both the gas phase and dimethylformamide (DMF), the experimental solvent(Karelson et al. 1996). Key descriptors including HOMO/LUMO energies, energy gap (ΔE), ionization potential (I), electron affinity (A), chemical hardness (η), softness (δ), electronegativity (χ), chemical potential (μ), and dipole moment (Cp) were derived to assess stability and reactivity (Table 3) (Akintemi et al. 2022; Brown & Simas 1982).

**Table 3:** Quantum chemical parameters

Cd	E_LUMO_	E_HOMO_	ΔE	A	I	η	δ	χ	C_p_	µ
8f_gas_	− 2.56	− 6.38	3.82	2.56	6.38	1.91	0.52	4.48	− 4.47	5.23
8f_dmf_	− 2.47	− 6.51	4.04	2.47	6.51	2.02	0.50	4.49	− 4.49	4.98
8j_gas_	− 2.50	− 6.25	3.75	2.50	6.25	1.87	0.53	4.37	− 4.37	5.10
8j_dmf_	− 2.46	− 6.40	3.94	2.46	6.39	1.97	0.51	4.42	− 4.42	4.97
8k_gas_	− 2.50	− 6.25	3.75	2.50	6.25	1.87	0.53	4.37	− 4.37	5.10
8k_dmf_	− 2.46	− 6.39	3.93	2.46	6.39	1.97	0.51	4.43	− 4.43	4.98
8l_gas_	− 3.28	− 6.54	3.25	3.28	6.54	1.63	0.62	4.91	− 4.91	7.41
8l_dmf_	− 3.34	− 6.52	3.18	3.34	6.52	1.59	0.63	4.93	− 4.93	7.64
Su_gas_	− 4.19	− 7.56	3.36	4.19	7.56	1.68	0.59	5.88	− 5.88	10.27
Su_dmf_	− 2.40	− 5.73	3.33	2.40	5.73	1.66	0.59	4.07	− 4.07	4.96

The energies of the lowest unoccupied molecular orbital (E_LUMO_) and highest occupied molecular orbital (E_HOMO_) are the fundamental parameters used to determine the other parameters according to the following equations:

2 $$ \Delta E = E_{LUMO} - E_{HOMO} $$

3 $$ I = - E_{HOMO} $$

4 $$ A = - E_{LUMO} $$

5 $$ \eta = \frac{\Delta E}{2} $$

6 $$ \delta = \frac{1}{\eta } $$

7 $$ \chi = \frac{{\left( {I + A} \right)}}{2} $$

8 $$ C_{P} = - \chi $$

Among the lead molecules, compound **8l** displayed the smallest energy gap (3.25 eV gas, 3.18 eV DMF), indicating the highest reactivity, followed closely by Sunitinib (3.36 eV and 3.33 eV). In contrast, **8f** showed increased ΔE in DMF, reflecting improved stability in the solvent. Similarly, ionization potentials and electron affinities supported these trends, with **8l** exhibiting high values in both environments (I = 6.54 eV, A = 3.28 eV), suggesting strong electron-donating and accepting capacity. Sunitinib displayed high values in gas phase but decreased significantly in DMF. Chemical hardness and softness further reinforced compound 8l’s reactivity. It recorded the lowest hardness (1.59–1.63 eV) and highest softness (0.62–0.63 eV), exceeding all other compounds, including Sunitinib. Additionally, electronegativity and chemical potential positioned **8l** as the most electron-attracting molecule among the leads, while Sunitinib showed higher electronegativity in gas phase but declined markedly in solvent. The dipole moment (Cp), indicative of molecular polarity, was highest in compound **8l** among the leads (7.41 Debye gas, 7.64 Debye DMF), suggesting a stronger interaction potential. Sunitinib showed the highest Cp overall in gas phase (10.27 Debye) but dropped significantly in DMF (4.96 Debye), indicating reduced polarity under solvated conditions [40–42](Sessa & Rahm 2022; Zaklika et al. 2022; Zhan et al. 2003). Generally, this comparative DFT analysis highlights that while Sunitinib demonstrates favorable stability, compound **8l** stands out due to its high reactivity, electron-attracting character, and significant polarity, particularly in the solvated phase. These properties reinforce **8l’s** potential as a strong Mcl-1 inhibitor and a promising candidate for further optimization in drug development (Politzer & Murray 2002).

#### Frontier molecular orbitals (FMO) and the molecular electrostatic potential (MEP)

**Fig. 8:**
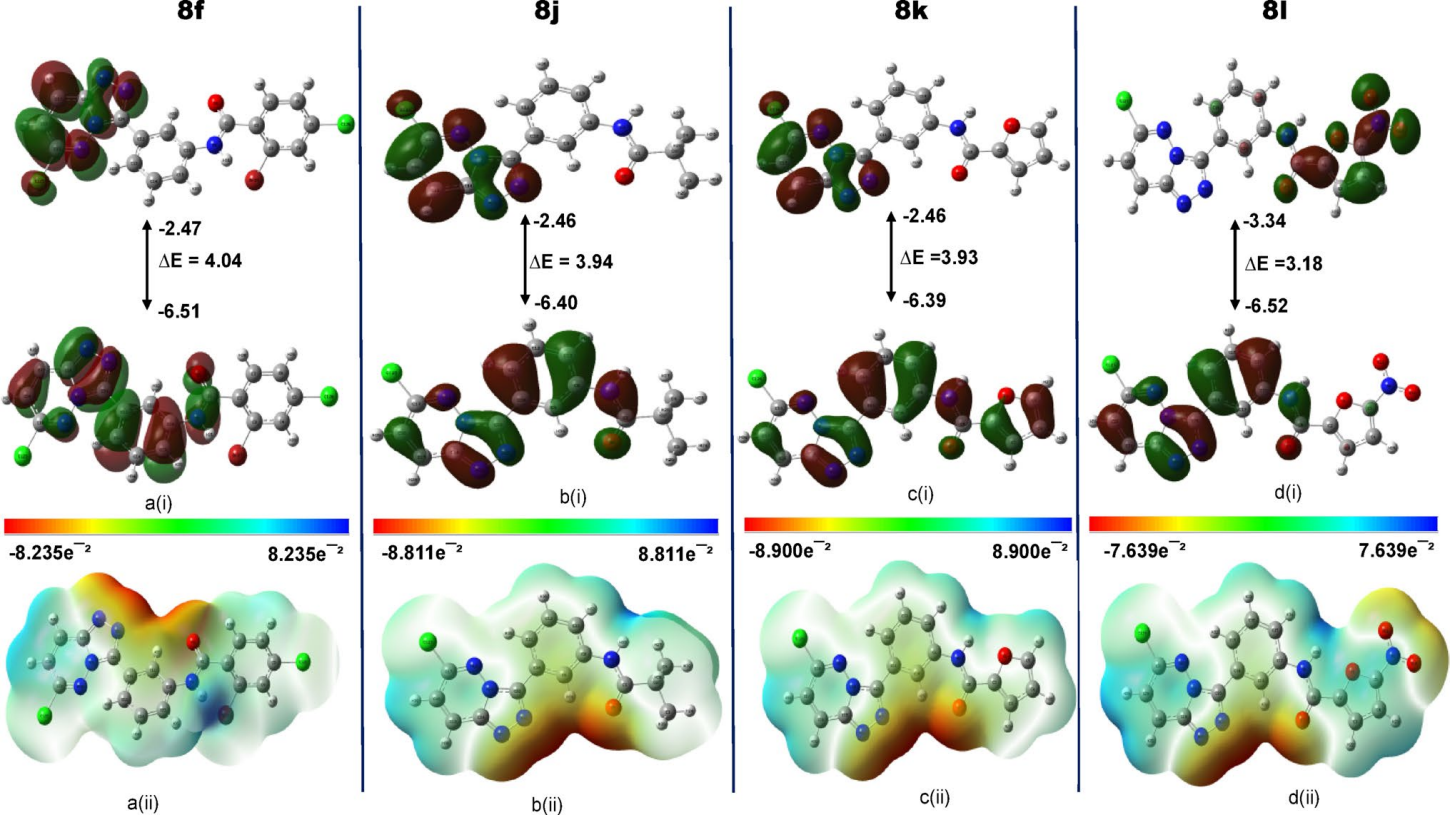
Frontier molecular orbital (FMO) diagrams and molecular electrostatic potential (MEP) maps

The frontier molecular orbitals (HOMO and LUMO) and molecular electrostatic potential (MEP) surfaces of compounds **8f**, **8j**, **8k**, and **8l** are presented in Fig. 8. These electronic descriptors provide valuable insight into charge distribution, electronic transitions, and potential interaction sites within each molecule. In compounds **8f** and **8j**, the LUMO was predominantly localized on the 6-chloro-[1,2,4]triazolo[4,3-b]pyridazine core, with **8f** showing minor extension into the benzamide moiety, while **8j** exhibited minimal contribution from its isobutyl-pivalamide side chain. The HOMO for both compounds was more delocalized, excluding regions such as the chlorinated phenyl and alkyl chains.

For **8k**, the LUMO remained concentrated on the triazolopyridazine core, with limited involvement from the phenylfuran group. It’s HOMO extended more broadly but excluded the chloro substituent and part of the nitro group. In contrast, **8l** exhibited a distinct profile: the LUMO was highly localized on the 5-nitrofuran-2-carboxamide, while the HOMO included contributions from both the triazolopyridazine and N-phenylacetamide regions, excluding the nitro-substituted ring. This orbital distribution supports **8l** enhanced reactivity and interaction potential. MEP analysis further elucidated molecular polarity and reactive sites (Braga et al. 2016; Politzer et al. 1985). As illustrated in Fig. 8, all compounds displayed blue regions corresponding to electron-deficient (nucleophilic) zones and red regions representing electron-rich (electrophilic) sites. These distributions align with expected interaction behavior in protein environments. Remarkably, compound **8l** showed a more intense red region around its NO₂ group, distinguishing it from the others. This localized negative potential indicates a highly reactive electrophilic site, reinforcing **8l** dual reactivity and strong binding potential. Such charge separation and polarity suggest that **8l** may engage more effectively in both nucleophilic and electrophilic interactions, enhancing its suitability for targeted therapeutic applications (Mjwara et al. 2025). The small HOMO–LUMO gap observed for **8l** (3.18 eV) confirms high chemical reactivity and a strong potential to interact with electron-deficient regions within the protein target. MEP maps further showed complementary nucleophilic/electrophilic regions, which support its binding to charged residues in the Mcl-1 binding pocket. This electronic signature aligns with its favorable docking and MD profile.

#### Drug-likeness, ADME, and toxicity profiling

The drug-likeness and ADME properties of compounds **8f**, **8j**, **8k**, and **8l**, along with the reference drug Sunitinib, were evaluated using Lipinski’s Rule of Five and QikProp predictions (Table 4) (Lipinski et al. 1997). All compounds satisfied Lipinski’s criteria, indicating potential for good oral bioavailability. Molecular weights ranged from 329.78 g/mol (**8j**) to 463.12 g/mol (**8f**), with all candidates remaining within the acceptable limits for hydrogen bond donors (≤ 5) and acceptors (≤ 10), and no violations of logP thresholds. Lipophilicity (QPlogPo/w) was highest for **8f** (4.65) and lowest for **8l** (2.23), indicating that the latter is more polar, which could affect membrane permeability. Blood–brain barrier permeability (QPlogBB) was low for all compounds, with **8f** being closest to the threshold (− 0.17), and **8l** exhibiting the lowest CNS exposure (− 1.89), suggesting limited neurotoxicity potential (Figueira et al. 2017). The cardiotoxicity risk, evaluated via QPlogHERG, was comparable across all compounds, with values below − 5, suggesting a low likelihood of hERG channel blockade (Lansu & Gentile 2013). Cellular permeability (QPPMDCK and QPPCaco) was highest for **8f**, followed by **8j** and Sunitinib, suggesting favorable intestinal absorption. In contrast, **8l** exhibited poor permeability in both models (Storchmannová et al. 2025). Solubility profiles (QPlogS) highlighted **8f** as poorly soluble (− 7.70), whereas **8j**, **8k**, and Sunitinib demonstrated moderate aqueous solubility (Palmer & Mitchell 2014). Predicted human oral absorption (%HOA) was highest for **8f** and **8j** (100%), while **8l** showed the lowest absorption (72.21%) due to limited solubility and permeability (Handa et al. 2023).

**Table 4:** Drug-likeness and ADME properties of the Lead compounds

Drug likeliness according to Lipinski’s Rule of Five Assessed by QikProp									ADME properties		
Compound	MW^a^	DonorHB^b^	AccptHB^c^	QPlogPo/w^d^	LRoF^e^	QPloHERG^f^	QPlogBB^g^	QPPMDCK^h^	QPPCaco^i^	QPlogS^j^	%HOA^k^
8f	463.12	1	5.50	4.65	0	− 6.54	− 0.17	6360.43	969.99	− 7.70	100
8j	329.78	1	5.50	3.40	0	− 6.65	− 0.39	1353.21	1084.44	− 4.62	100
8k	339.74	1	6.00	2.81	0	− 6.34	− 0.66	700.20	586.90	− 4.93	92.98
8l	384.73	1	7.00	2.23	0	− 6.49	− 1.89	62.77	63.02	− 5.43	72.21
Sunitinib	398.47	2	6.00	3.91	0	− 6.58	− 0.61	190.48	218.17	− 4.65	91.74

^a^Molecular Weight (Acceptable Range: < 500)

^b^Hydrogen Bond Donor (Acceptable Range: < 5)

^c^Hydrogen Bond Acceptor (Acceptable Range: < 10)

^d^Predicted Octanol/Water Partition Coefficient (log P) (Acceptable Range: − 2.0 to 6.5)

^e^Lipinski’s Rule of Five-LRoF (No Violation)

^f^Predicted QPlogHERG parameter (Acceptable Range: < 5 is lower likelihood and > 5 is higher lower likelihood

^g^Predicted Brain/Blood Partition Coefficient (Acceptable Range: − 3.0 to 1.2)

^h^Predicted apparent MDCK cell permeability in nm/s (acceptable range: < 25 is poor and > 500 is good)

^i^Predicted Caco-2 Cell Permeability (nm/s) (Acceptable Range: < 25, poor; > 500, good)

^j^Predicted Aqueous Solubility (mol/L) (Acceptable Range: − 6.5 to 0.5)

^k^Percentage of human oral absorption (HOA) (Acceptable Range: < 25%, poor; > 80%, high)

The significance of these findings lies in the trade-off between pharmacokinetic properties and target-binding affinity. While **8f** displays superior solubility, permeability, and absorption, it lacks the strong binding affinity and dynamic stability of **8l**, which emerged as the most potent Mcl-1 inhibitor from docking and MM/GBSA calculations. This highlights **8l** as a lead compound requiring further optimization to improve solubility and permeability while maintaining its favorable pharmacodynamic profile. These insights will guide structural refinements to enhance both efficacy and bioavailability. All ADME simulations utilized the OPLS4 force field (Huang et al. 2005) and TIP4P water model (Jorgensen et al. 1983), which are known for accurate representation of solvation and conformational space in drug-likeness predictions.

Toxicity profiling using ProTox 3.0 (Table 5) revealed that all compounds fall under toxicity class IV (300 < LD50 ≤ 2000 mg/kg), indicating they are harmful if swallowed. Most compounds were predicted to be hepatotoxic, with **8l** showing additional risks for carcinogenicity and mutagenicity. In contrast, Sunitinib was non-hepatotoxic but showed immunotoxic potential (Banerjee et al. 2018). These toxicity results are consistent with the observed pharmacokinetic trends and point toward compound **8l** dual need for optimization: enhancing ADME profiles while monitoring toxicity liabilities. The hepatotoxicity of **8l**, coupled with its mutagenicity risk, underscores the importance of medicinal chemistry efforts to reduce toxicity while preserving its potent Mcl-1 binding capacity. These predictions serve as early flags to prioritize safer analogs in future in vitro validation. We acknowledge that the study’s computational nature lacks experimental confirmation at this stage. However, the integration of robust modeling strategies combining molecular docking, MM/GBSA, molecular dynamics, and quantum chemical analyses provides a validated predictive framework for early-stage drug discovery. Previous studies have demonstrated strong correlations between in silico predictions and in vitro outcomes, particularly for structurally similar scaffolds. As such, our findings serve as a valuable hypothesis-generating step, guiding compound prioritization and de-risking subsequent experimental efforts. We anticipate that future experimental work will be directed toward validating compound **8l** Mcl-1 inhibition, bioavailability, and safety.

**Table 5:** Toxicity Prediction

Compd	Hepato	Carcino	Immuno	Mutagen	LD50(mg/kg)	Toxicity class
8f	Active	Inactive	Inactive	Inactive	1000	4
8j	Active	Inactive	Inactive	Inactive	560	4
8k	Active	Inactive	Inactive	Inactive	2000	4
8l	Active	Active	Inactive	Active	600	4
Su	Inactive	Inactive	Active	Inactive	500	4

*Hepato* hepatotoxicity, *Carcino* carcinogenicity, *Immuno* immunotoxicity, *Mutagen* mutagenicity, *Cytoto* cytotoxicity, Class I: fatal if swallowed (LD50 ≤ 5), Class II: fatal if swallowed (5 < LD50 ≤ 50), Class III: toxic if swallowed (50 < LD50 ≤ 300), Class IV: harmful if swallowed (300 < LD50 ≤ 2000), Class V: may be harmful if swallowed (2000 < LD50 ≤ 5000), Class VI: non-toxic (LD50 > 5000)

The selectivity concerns associated with Mcl-1 inhibitors were addressed in part by detailed binding energy decompositions and molecular dynamics simulations, which revealed unique non-covalent interactions and favorable packing patterns specific to Mcl-1. These findings suggest that triazolo[4,3-b]pyridazine derivatives, particularly **8l** and **8k**, may exploit binding site features that contribute to selectivity. Nonetheless, future studies involving broader target panels are necessary to validate these preliminary indications. Overall, the integration of structural, dynamic, electronic, and pharmacokinetic evaluations reinforces compound 8l as a promising lead scaffold. The concordance between docking, MD, DFT, and prior experimental data supports its advancement toward optimization. These insights provide a foundation for rationally designing analogs with improved bioavailability and reduced toxicity. Although compound **8f** exhibited the most favorable GlideScore (− 8.91 kcal/mol) and superior ADME properties, compound 8l was prioritized based on its consistent thermodynamic stability, strong MM/GBSA binding energy (− 58.96 kcal/mol), and favorable electronic profile. Molecular dynamics simulations of the 8l-Mcl-1 complex revealed stable RMSD and RMSF trajectories, indicating a robust binding conformation over time.

Furthermore, its HOMO–LUMO gap of 3.18 eV signified high electronic reactivity and potential for strong target interaction. While ADME limitations such as moderate solubility and permeability were noted, these can be improved through future scaffold refinements. Importantly, the compound’s prior in vitro antiproliferative activity (IC₅₀ = 1.5 µM) against MV4-11 AML cells (Pathan et al. 2023) reinforces its candidacy for further development. Given the dual need for improved pharmacokinetics and experimental confirmation, planned validation studies will focus on testing compound 8l in relevant AML models. Specifically, we will evaluate its cytotoxicity using cell viability assays and confirm Mcl-1 binding via biochemical methods, such as fluorescence polarization or SPR. Thus, compound **8l** represents a structurally and energetically superior lead scaffold that merits optimization to enhance its drug-likeness without compromising potency.

## Conclusion

This study employed a comprehensive computational strategy encompassing molecular docking, molecular dynamics (MD) simulations, MM/GBSA free energy calculations, DFT-based quantum chemical analysis, and ADMET/toxicity profiling to evaluate a series of 1,2,4-triazolo[4,3-b]pyridazine derivatives as potential Mcl-1 inhibitors for Acute Myeloid Leukemia (AML). Among the compounds assessed, compound **8l** emerged as the most promising lead, exhibiting the strongest binding affinity (ΔG_bind_ = − 58.96 kcal/mol), high electronic reactivity (ΔE = 3.18 eV in DMF), and potent experimental antiproliferative activity (IC₅₀ = 1.5 µM), outperforming the reference drug sunitinib. MD simulations confirmed the structural stability of the **8l**-Mcl-1 complex, while frontier orbital and electrostatic analyses highlighted favorable charge distributions and dual-reactivity profiles. Although ADMET analysis revealed limitations such as poor solubility and moderate permeability, these are offset by the compound’s strong pharmacodynamic profile. Toxicity predictions indicated manageable safety concerns, with hepatotoxicity and mutagenicity flagged for future attention. While these findings are robust, experimental validation remains essential to confirm the therapeutic relevance of the identified compounds fully. In light of these promising computational findings, we are initiating experimental validation studies. These include biochemical assays such as surface plasmon resonance (SPR) and enzyme inhibition kinetics to confirm direct binding of compound **8l** to Mcl-1, as well as MTT and apoptosis assays using AML cell lines (MV4-11, HL-60) to confirm antiproliferative effects. These efforts will provide mechanistic and translational validation of the computational predictions presented herein. Structural refinements will also be pursued to improve solubility, reduce toxicity risks, and enhance drug-likeness without compromising potency. In all, this study positions compound **8l** as a viable scaffold for further development and underscores the utility of integrated computational approaches in accelerating the discovery of selective anti-AML therapeutics targeting Mcl-1.

## Supplementary Information

Below is the link to the electronic supplementary material.

Supplementary Material 1

## Data Availability

No datasets were generated or analysed during the current study.
