# Supplementary material for: Targeting Acute Myeloid Leukemia with 1,2,4-triazolo[4,3-b]pyridazine derivatives: a molecular docking, dynamics, and ADMET approach
Source: In Silico Pharmacol. 2025 Sep 17;13(3):135. doi: 10.1007/s40203-025-00418-1 (PMC12443659; doi:10.1007/s40203-025-00418-1)
Supplement: Supplementary file 1 — Supplementary Material 1 [file 40203_2025_418_MOESM1_ESM.docx]

**Supplementary Material**

**Targeting Acute Myeloid Leukemia with 1,2,4-Triazolo[4,3-b]pyridazine Derivatives: A Molecular Docking, Dynamics, and ADMET Approach**

Vincent A. Obakachi^1^*, Krishna K. Govender^1^*, Penny P. Govender^1^

 **S1**


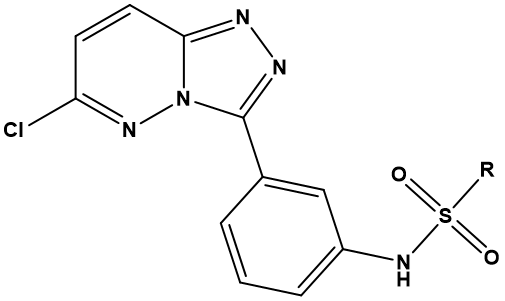


**7a - 7d 8a -8m 9a -9i**

| SN | Compounds | X | R | Docking Score |
| --- | --- | --- | --- | --- |
| 1 | 7a | S | 4Cl | -8.43 |
| 2 | 7b | S | 4F | -8.14 |
| 3 | 7c | O | 4Br | -7.86 |
| 4 | 7d | O | 4Br5CF_3_ | -8.49 |
| 5 | 8a | - | Ph4CH_3_ | -8.49 |
| 6 | 8b | - | Ph3OCH_3_ | -7.99 |
| 7 | 8c | - | Ph2Br4,5OCH_3_ | -8.40 |
| 8 | 8d | - | Ph3Cl | -8.03 |
| 9 | 8e | - | Ph4Br | -8.32 |
| 10 | 8f | - | Ph2Br4Cl | -8.91 |
| 11 | 8g | - | Ph2Br | -8.25 |
| 12 | 8h | - | Ph4I | -7.05 |
| 13 | 8i | - | Ph4F | -8.46 |
| 14 | 8j | - | 3CH_3_ | -8.85 |
| 15 | 8k | - | 1-furyl | -8.75 |
| 16 | 8l | - | 5-nitro-2-furyl | -8.81 |
| 17 | 8m | - | 2N-Ph | -8.44 |
| 18 | 9a | - | 4CH_3_ | -6.90 |
| 19 | 9b | - | 4OCH_3_ | -6.58 |
| 20 | 9c | - | 4Ph | -7.36 |
| 21 | 9d | - | Ph3,4Cl | -7.42 |
| 22 | 9e | - | Ph4Br | -7.32 |
| 23 | 9f | - | Ph4I | -6.72 |
| 24 | 9g | - | Ph3NO_3_ | -8.36 |
| 25 | 9h | - | Ph4NO_3_ | -6.14 |
| 26 | 9i | - | Ph4F5Cl | -7.45 |
| 27 | Sunitinib | - | - | -8.74 |
